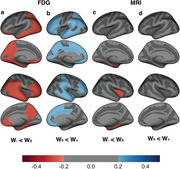# Integration of Clinical and Biological Staging Scheme for Alzheimer's Disease

**DOI:** 10.1002/alz70856_099704

**Published:** 2025-12-24

**Authors:** Young‐gun Lee

**Affiliations:** ^1^ Ilsan Paik Hospital, Inje University College of Meidicine, Goyang, Gyeonggi, Korea, Republic of (South)

## Abstract

**Background:**

Integrating biological and clinical staging could reveal factors underlying Alzheimer's disease (AD) heterogeneity.

**Method:**

In 193 participants from the Alzheimer's Disease Neuroimaging Initiative, the discrepancy between clinical stage (cognition) and biological stage (tau deposition) was measured using standardized residuals (W‐score) to classify groups: concordant stage (W_0_), worse clinical stage (W_‐_), and better clinical stage (W_+_).

**Result:**

The W_‐_ group showed faster cognitive decline and higher progression risk (HR = 2.40, 95% CI = 1.20 – 4.83), while the W_+_ group had lower progression risk (HR = 0.43, 95% CI = 0.19 – 0.96). Co‐pathology burden was correlated with W‐scores, and the W_‐_ group had higher ratio of cerebrospinal α‐synuclein positivity. Lower brain metabolism was observed in the W_‐_ group, whereas higher brain metabolism was noted in the W_+_ group compared to the W_0_ group.

**Conclusion:**

Integrated staging scheme has clinical implications on predicting AD prognosis and underlying co‐pathology.